# Health Literacy and Quality of Life in Patients With Type 1 Diabetes Mellitus

**DOI:** 10.7759/cureus.10860

**Published:** 2020-10-09

**Authors:** İrfan Esen, Selin Aktürk Esen

**Affiliations:** 1 Internal Medicine, Yildirim Beyazit University Yenimahalle Research and Training Hospital, Ankara, TUR; 2 Medical Oncology, Health of Science Ankara City Hospital, Ankara, TUR

**Keywords:** audit of diabetes dependent quality of life, type 1 diabetes mellitus, diabetic complications, health literacy, newest vital sign

## Abstract

Aim

The aim of this study was to investigate the relationship between health literacy (HL) and quality of life (QoL) in type 1 diabetes mellitus (DM) patients.

Method

This study was conducted between February 2020 and May 2020 at the University of Health Sciences Bursa Yuksek Ihtisas Training and Research Hospital, in Turkey. A total of 155 patients with type 1 DM between the ages of 18-65 were included in the study. QoL was evaluated with the Audit of Diabetes Dependent QoL questionnaire and HL was evaluated with the Newest Vital Sign (NVS) questionnaire and obtained results were compared.

Results

The weighted impact score for the overall QoL scale was higher for patients who did not have complications than those with complications (p=0.004). Retinopathy and nephropathy were higher in the group with low HL (p=<0.001; p=0.032; p=0.012, respectively). The weighted impact score for the overall QoL scale was lower in married individuals (p=0.040) and it was higher for high school and above education levels than those with lower education levels (p=0.004). The sex life weighted impact score was higher in the group with DM less than 10 years (p=0.045).

Conclusion

Patients with high HL status are more adaptable to their physician's recommendations, less frequent complications will occur in these patients and the QoL of the patients will be better in the absence of complications.

## Introduction

Diabetes mellitus (DM) is a public health problem that causes disability, death, loss of workforce and leads to a lower quality of life (QoL) [1]. There are approximately 424.9 million DM patients worldwide, and it is estimated to reach 628.6 million in 2045 [2]. DM is a life-threatening disease with retinopathy, neuropathy, nephropathy, and cardiovascular complications [3].

Studies show that the quality QoL of DM patients is lower than those without the disease. DM type, complications, social environment, insulin use, psychological factors, low level of education, and insufficiency of disease information are thought to affect the QoL negatively [4]. QoL for type 1 DM patients has been investigated in many studies [5]. Studies showed that the topics that most affect the QoL for type 1 DM patients are the freedom of eating and worrying about their future [6]. All these issues are associated with microvascular and macrovascular complications resulting from patient failure in blood glucose regulation. Patients with high QoL are expected to have good disease outcomes.

Health literacy (HL) skills can be summarized as having a dialogue and discussion with the individual about his/her illness, reading and interpreting health information, calculating drug timing and dosage, using medical tools for personal or family health care, and making decisions about participating in research studies [7]. In DM cases, HL has a positive effect on patient follow-up results [8-10]. Blood glucose regulation and diabetic complications are known to be in parallel with HL in DM patients [8].

The present study aimed to investigate the relationship between HL and QoL in patients with type 1 diabetes and its reflection on patient outcomes.

## Materials and methods

Study design and data source

This cross-sectional study was conducted between February 2020 and May 2020 at the University of Health Sciences Bursa Yuksek Ihtisas Training and Research Hospital in Turkey. Patients aged less than 18 years and over 65 years and diagnosed with type 1 diabetes for less than six months were excluded. The patients were informed about the research, and written consent was obtained. One hundred and fifty-five patients were included. The study was approved by the University of Health Sciences Bursa Yuksek Ihtisas Training and Research Hospital ethics committee with decision number 2011-KAEK-25 2019/01-20.

Scales

Participants completed a form that included age, gender, marital status, educational status, occupation, health insurance, economic income, presence of diabetic complications, and comorbid disease information.

QoL was evaluated with the Audit of Diabetes Dependent QoL (ADDQoL) questionnaire. This scale is a diabetes-specific scale used in many countries since 1994 [11-13]. It has 13 specific domains and two overview elements (one for general and one for diabetes-specific QoL). Participants receive both impact (between -3 and +3) and importance (between 0 and 3) points for each of the 13 specific domains. To find the weighted impact score, these two values are multiplied (between -9 and +9). An average weighted impact score is derived by totaling the weighted impact scores for each domain and dividing by the number of the applicable domains. If the participant marked that the question is not applicable for her/him, this question scored zero and did not contribute to the total score. The ADDQoL questionnaire is gradually becoming useful for treatment, intervention, self-management programs, and clinical trials of DM [14]. The internal consistency of the ADDQoL Turkish version was found to be high (Cronbach's α=0.90-0.91), which indicates that the Turkish version of this instrument is reliable [4].

Newest Vital Sign (NVS) questionnaire is a practical instrument that tests HL [15]. A nutrition label from an ice cream box was given to the patients to read carefully. The patients were then given six questions and asked to respond on the form. For each correct answer, patients were given one point. A total score lower than four indicates limited HL. This survey is related to mathematical ability and reading comprehension. The validity of the Turkish version of the NVS scale was reported by Ozdemir et al. [16].

Statistical analysis

A post hoc power analysis was conducted using a small effect size based upon findings of the present study. The small effect size was obtained by comparing the average weighted impact scores between NVS groups. Using this effect size (d=0.21) with a sample size of 155 participants, achieved power was estimated as 80% at a significance level of α=0.05. Kolmogorov-Smirnov test was used to assess whether the variables followed a normal distribution. Variables were reported as mean ± standard deviation (minimum: maximum) or median (minimum: maximum) values. According to normality test results, the Mann-Whitney U test was used to perform between-group comparisons. Categorical variables were compared by Chi-square test or Fisher's exact test. In order to estimate the sensitivity and specificity of weighted impact score values for predicting the absence of complications, receiver operator characteristic (ROC) curve analysis was performed. The area under the ROC curve value with 95% confidence intervals (CIs) was reported. SPSS® version 23.0 (IBM Inc, Armonk, USA) and MedCalc® Statistical Software trial version 16.4.3 (MedCalc Software Ltd, Ostend, Belgium) were used. A p-value of less than 0.05 was considered statistically significant.

## Results

The mean duration of DM in participated patients was 11 (1:37) years. DM complications were present in 46.45% of patients; 27.09% (n=42) of the patients had other diseases (asthma, hyperlipidemia, migraine, etc.). Socio-demographic characteristics are summarized in Table 1.

**Table 1:** Socio-demographic characteristics of the patients

Characteristic	n (%)
Gender	
Female	93 (60.0%)
Male	62 (40.0%)
Age	
≥40 years	27 (17.4%)
<40 years	128 (82.6%)
Marital status	
Married	89 (57.4%)
Other	66 (42.6%)
Education	
< High school	58 (37.4%)
≥ High school	97 (62.6%)
Monthly income ($)	
>400	49 (31.6%)
≤400	106 (68.4%)
Insurance	
Yes	139 (89.7)
No	16 (10.3%)
Occupation	
Working	91 (58.7%)
Retired - not working	64 (41.3%)
Complications	
Yes	72 (46.5%)
No	83 (53.5%)
Comorbidity	
Yes	42 (27.1%)
No	113 (72.9%)
Diabetes duration (years)	
<10 years	20 (12.9%)
≥10 years	135 (87.1%)

The relationship between NVS and QoL is shown in Table 2. In the group with a high NVS, diabetes has the most negative impact on the enjoyment of the food subscale (impact score = -3). The impact, importance, and weighted impact scores of the ADDQoL family relationships subscale were found to be higher in the group with a high HL level (p=0.009; p=0.021; p=0.008, respectively). The impact and weighted impact scores of the ADDQoL sex life subscale were found to be higher in the group with a high HL level (p=0.015; p=0.031, respectively).

**Table 2:** NVS and quality of life scale intersection table ADDQoL - Audit of Diabetes Dependent Quality of Life; NVS -  Newest Vital Sign

ADDQoL-13	Score	High NVS (n=58): a total score of ≥ 4	Low NVS (n=97): a total score of < 4	p-value
Average	Impact	-1.31 (-3:0.85)	-1.62 (-3:0.15)	0.128
	Importance	2.19 (0:3)	2.15 (0.46:3)	0.299
	Weighted impact	-3.39 (-8.77:1.92)	-4 (-9:0.08)	0.220
Employment/career	Impact	-1 (-3:2)	-2 (-3:3)	0.101
	Importance	2 (0:3)	2 (-1:3)	0.347
	Weighted impact	-2 (-9:4)	-3 (-9:9)	0.234
Social life	Impact	-1 (-3:2)	-1 (-3:1)	0.566
	Importance	2 (0:3)	2 (0:3)	0.714
	Weighted impact	-2.5 (-9:4)	-3 (-9:1)	0.637
Family relationships	Impact	0 (-3:3)	-1 (-3:3)	0.009
	Importance	2 (0:3)	3 (0:3)	0.021
	Weighted impact	0 (-9:2)	-3 (-9:3)	0.008
Friends	Impact	0 (-3:3)	0 (-3:2)	0.377
	Importance	2 (0:3)	2 (0:3)	0.432
	Weighted impact	0 (-9:4)	0 (-9:3)	0.396
Sex life	Impact	0 (-3:3)	-1 (-3:3)	0.015
	Importance	2 (0:3)	2 (-1:3)	0.051
	Weighted impact	0 (-9:9)	-1 (-9:3)	0.031
Sport/leisure	Impact	-1 (-3:2)	-2 (-3:2)	0.397
	Importance	2 (0:3)	2 (0:3)	0.529
	Weighted impact	-2 (-9:4)	-3 (-9:4)	0.984
Travel	Impact	-2 (-3:2)	-2 (-3:3)	0.405
	Importance	2 (0:3)	2 (0:3)	0.555
	Weighted impact	-4 (-9:4)	-3 (-9:6)	0.388
Future (own)	Impact	-2 (-3:2)	-2 (-3:3)	0.517
	Importance	3 (0:3)	3 (0:3)	0.330
	Weighted impact	-6 (-9:6)	-6 (-9:9)	0.413
Future of family	Impact	-2 (-3:3)	-3 (-3:3)	0.206
	Importance	3 (0:3)	3 (0:3)	0.068
	Weighted impact	-6 (-9:9)	-6 (-9:9)	0.132
Motivation	Impact	-2 (-3:1)	-2 (-3:3)	0.415
	Importance	2 (0:3)	3 (0:3)	0.085
	Weighted impact	-4 (-9:2)	-6 (-9:9)	0.431
Physical activities	Impact	-2 (-3:1)	-3 (-3:0)	0.191
	Importance	2.5 (0:3)	3 (0:3)	0.371
	Weighted impact	-6 (-9:3)	-6 (-9:0)	0.267
Others fussing	Impact	0 (-3:2)	0 (-3:2)	0.056
	Importance	1 (0:3)	2 (0:3)	0.442
	Weighted impact	0 (-9:2)	-1 (-9:6)	0.019
Enjoyment of food	Impact	-3 (-3:3)	-3 (-3:0)	0.289
	Importance	3 (0:3)	3 (0:3)	0.627
	Weighted impact	-9 (-9:3)	-6 (-9:0)	0.270

In the groups with retinopathy, neuropathy, and nephropathy, the importance score for the overall QoL scale was found to be higher than in the groups without these complications (p=0.013; p=0.005; p=0.042, respectively). In the groups without retinopathy and neuropathy, the impact score for the overall QoL scale was higher compared to the groups in which these complications were observed (p=0.022; p=0.035, respectively) (Table 3).

**Table 3:** Relationship between the quality of life and diabetic complications

		Impact score	Importance score	Weighted impact score
Complications (retinopathy + neuropathy + nephropathy)	Yes (n=72)	-1.81 (-3:0.69)	2.31 (0.46:3)	-4.38 (-9:0)
	No (n=83)	-1.08 (-3:0.85)	2.08 (0:3)	-2.62 (-9:1.92)
	p-value	0.071	0.004	<0.001
Retinopathy	Yes (n=36)	-1.85 (-3:0)	2.35 (0.62:3)	-4.54 (-9:-0.15)
	No (n=119)	-1.23 (-3:0.85)	2.08 (0:3)	-3.15 (-9:1.92)
	p-value	0.022	0.013	0.060
Neuropathy	Yes (n=15)	-2.08 (-3:-0.38)	2.54 (0.46:3)	-5.62 (-8.77:-0.92)
	No (n=140)	-1.38 (-3:0.85)	2.08 (0:3)	-3.42 (-9:1.92)
	p-value	0.035	0.005	0.157
Nephropathy	Yes (n=48)	-1.69 (-3:0.69)	2.23 (0.54:3)	-4.23 (-9:0)
	No (n=107)	-1.23 (-3:0.85)	2.15 (0:3)	-3.15 (-9:1.92)
	p-value	0.427	0.042	0.073
Cardiovascular disease	Yes (n=5)	-2.08 (-3:0)	2.38 (1.15:2.92)	-5.31 (-8.77:0)
	No (n=150)	-1.46 (-3:0.85)	2.15 (0:3)	-3.69 (-9:1.92)
	p-value	0.619	0.883	0.423
Additional disease	Yes (n=42)	-1.66 (-3:0.15)	2.31 (0:3)	-4.12 (-8.77:0)
	No (n=113)	-1.38 (-3:0.85)	2.08 (0.23:3)	-3.54 (-9:1.92)
	p-value	0.287	0.207	0.138

Complications, retinopathy and nephropathy were higher in the group with low HL (p<0.001; p=0.032; p=0.012, respectively). There was no difference between the HL groups according to the prevalence of neuropathy, cardiovascular disease, and other comorbidities (Table 4).

**Table 4:** Relationship between health literacy and complications

	High NVS (n=58)	Low NVS (n=97)	p-value
Complications			
Yes	16 (27.6%)	56 (57.7%)	<0.001
No	42 (72.4%)	41 (42.3%)	
Retinopathy			
Yes	8 (13.8%)	28 (28.9%)	0.032
No	50 (86.2%)	69 (71.1%)	
Neuropathy			
Yes	3 (5.2%)	12 (12.4%)	0.142
No	55 (94.8%)	85 (87.6%)	
Nephropathy			
Yes	11 (19.0%)	37 (38.1%)	0.012
No	47 (81.0%)	60 (61.9%)	
Cardiovascular disease			
Yes	2 (3.4%)	3 (3.1%)	1.00
No	56 (96.6%)	94 (96.9%)	
Additional disease			
Yes	12 (20.7%)	30 (30.9%)	0.165
No	46 (79.3%)	67 (69.1%)	

Average weighted impact scores by socio-economic characteristics of the participants are shown in Table 5. The weighted impact score of the sport/leisure subscale was higher in the <40 age group (p=0.012). The weighted impact score for the overall QoL scale was lower for married individuals (p=0.040). The weighted impact score of the QoL scale was higher for high school and above education levels than those with lower education levels (p=0.004). In the group with an income level of $400 and above, only the social life subscale's weighted impact score was higher (p=0.028). Others fussing subscale's weighted impact score was higher for those with insurance (p=0.022). The travel subscale's weighted impact score was higher for employed individuals (p=0.030). The weighted impact score for the overall QoL scale was higher for patients who did not have complications compared to those with complications (p=0.004). Social life (p=0.020) and others fussing (p=0.007) subscales' weighted impact scores were higher in the group without comorbidity. The sex life subscale's weighted impact score was higher in the group with DM less than 10 years (p=0.045).

**Table 5:** Average weighted impact scores according to socio-economic characteristics of the participants

Average weighted impact score	Median (min: max)	p-value	Subscales with significant difference
Gender			
Female (n=93)	-3.15 (-9:-1.92)	0.165	Social life, sex life
Male (n=62)	-4.15 (-8.54:0)		
Age (year)			
≥40 (n=27)	-4.77 (-9:0.08)	0.086	Sport/leisure
<40 (n=128)	-3.58 (-9:1.92)		
Marital status			
Married (n=89)	-4.15 (-9:1.92)	0.040	Family relationships, sex life, future of family, physical activities
Other (n=66)	-2.62 (-8.77:0.08)		
Education			
< High school	-4.69 (-9:0)	0.004	Employment career, family relationships, travel, future (own), future of family, motivation, physical activities, others fussing
≥ High school (n=97)	-3.15 (-7.77:1.92)		
Monthly income ($)			
> 400 (n=49)	-3.31 (-9:0)	0.131	Social life
≤ 400 (n=106)	-3.96 (-9:1.92)		
Insurance			
Yes (n=139)	-3.62 (-9:1.92)	0.162	Others fussing
No (n=16)	-4.96 (-7.31:0)		
Occupation			
Working (n=91)	-3.31 (-8.54:1.92)	0.433	Travel
Retired - not working (n=64)	-3.92 (-9:0.08)		
Complications			
Yes (n=72)	-4.38 (-9:0)	0.004	Employment career, social life, family relationships, friends, sex life, travel, motivation
No (n=83)	-2.62 (-9:1.92)		
Comorbidity			
Yes (n=42)	-4.12 (-8.77:0)	0.207	Social life, others fussing
No (n=113)	-3.54 (-9:1.92)		
Diabetes duration (years)			
<10 (n=20)	-2.58 (-7.77:-0.31)	0.286	Sex life
≥10 (n=135)	-3.77 (-9:1.92)		

**Figure 1:**
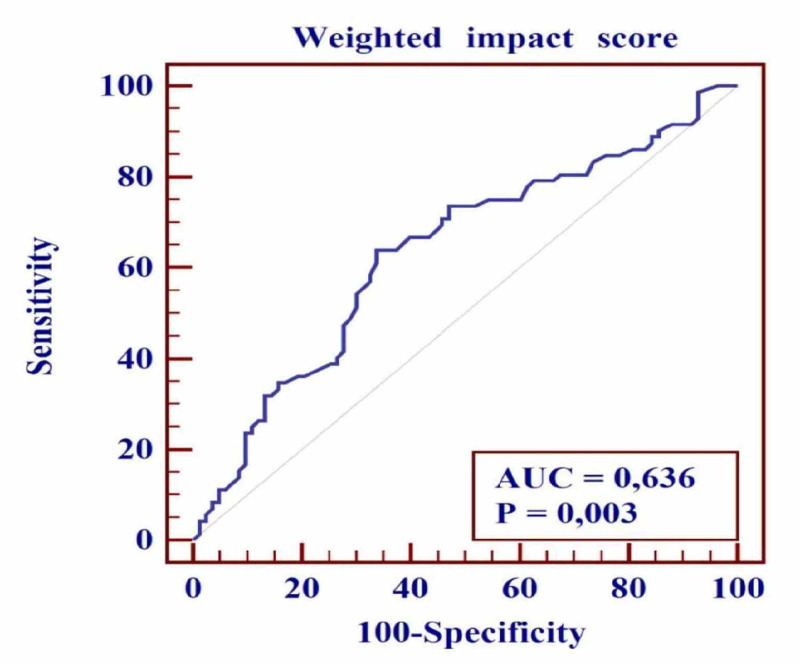
Receiver operator characteristic (ROC) curve for determining the absence of complications The area under the curve (AUC) for weighted impact scores is 0.64 (95% CI: 0.56-0.71) with p=0.003.

Receiver operator characteristic curve analysis was performed to estimate the sensitivity and specificity of the weighted impact score for predicting the absence of complications, and the cut-off point for the weighted impact score was determined as ≤-3.92 (Figure 1). The area under the curve for the weighted impact score was 0.64 (sensitivity 63.89%, specificity 66.27%, 95% CI: 0.56-0.71; p = 0.003), showing that a weighted impact score value ≤ -3.92 was significantly related to an increased risk of the absence of complications.

## Discussion

This study showed that both HL and QoL were associated with DM complications. Previous studies on HL and DM have shown an association of diabetic complications with low HL [8, 17]. Health literacy includes access of patients to treatment services and the protection and development of patients' health. Patients with low HL may have difficulty accessing treatment services when DM develops. We observed that complications have a negative effect on QoL in this study. There was a strong correlation between the presence of complications and the QoL weighted impact score. In these patients, retinopathy and subsequent blindness may impair the QoL of patients and their families. Nephropathy and subsequent chronic renal failure may result in dialysis treatment or even loss of the patient. Cardiac complications and dyspnea reduce the functional capacity of patients and adversely affect QoL. The development of complications may also cause a loss of workforce. Due to retinopathy, nephropathy, or other complications, patients with DM may become unable to work. Economic collapse may further impair people's QoL.

Periodic complication screening of patients may prevent both complications and deterioration in QoL. Besides this, the relationship of periodic examinations with HL has been demonstrated in previous studies [18, 19]. Patients with high HL follow the doctor's instructions better and give more importance to controls. Perhaps this relationship between HL and QoL is related to patients' compliance with the physician's instructions. In the previous studies, a negative correlation was also found between HL and hemoglobin A1C levels [8, 17, 20, 21]. Similarly, patients with low HL were shown to use higher doses of insulin [22]. This deterioration in the course of the disease in patients with low HL levels can lead to the development of the complications more easily and can lead to low QoL. So this situation may create a vicious cycle. 

We detected that in type 1 DM, patients with low HL, namely family relationship, sex life, and others fussing, which are subscales of QoL, were worse than the group with high HL. Bad sex life in men can be explained by erectile dysfunction as a result of poorly controlled DM [23, 24]. Vascular complications of DM in women can lead to loss of libido [25]. Therefore, the relationship between HL and complications seems to affect QoL indirectly. The deterioration of sex life adversely affects the partner and family relationship.

In the present study, QoL was worse in people with low education levels and married people. A study showed that education levels might be associated with low HL in general, but not in all cases [26]. An increase in education levels can also facilitate access to information about the disease. Thus, patients can cope with the disease more consciously. Decreasing uncertainty about the disease will increase QoL.

There were some limitations of our study. First, this study was performed with patients who applied to a tertiary hospital. It does not give us an idea about patients who never applied to the hospital or rarely visited a physician. In this study, patient compliance with physician recommendations was not evaluated. Carrying out control visits may be a major factor in the relationship between HL and QoL in type 1 DM patients.

## Conclusions

In type 1 DM patients, low QoL levels are associated with low HL levels and increased complications. This relationship can be explained by the incompatibility of people with low HL to the doctor's recommendations. Assuming that patients with high HL are more adaptable to their physician's recommendations, less frequent complications will occur in these patients. With the reduction of complications, the QoL in these patients will increase. Considering all these factors, type 1 DM patients should be trained at every opportunity to improve HL and QoL and to reduce complications.
